# Cerebral hemodynamics and safety of ciprofol in laparoscopic bariatric surgery: a parallel, double-blind, randomized controlled clinical trial

**DOI:** 10.3389/fmed.2025.1726287

**Published:** 2025-12-18

**Authors:** Qichen Luo, Shuqing Liang, Xiaoli Wang, Zhongyou Tian, Yalan Li

**Affiliations:** 1 Department of Anesthesiology, The First Affiliated Hospital of Jinan University, Guangzhou, Guangdong Province, China; 2 Jinan University, Guangzhou, Guangdong Province, China

**Keywords:** cerebral flow velocity, ciprofol, hemodynamic, laparoscopic bariatric surgery, propofol

## Abstract

**Background:**

Obesity has become a global health crisis. Laparoscopic bariatric surgery, a commonly used method for treating obesity, can significantly alter intracranial hemodynamics due to surgical positioning and pneumoperitoneum pressure, thereby increasing the likelihood of perioperative complications in obese individuals. Ciprofol, a novel propofol analog, offers advantages such as reduced side effects and enhanced stability, but its effects on brain blood flow regulation in obese patients are not yet fully understood. This clinical trial was designed to validate its superior efficacy in preserving cerebral perfusion and maintaining hemodynamic stability.

**Methods:**

The trial was structured as a randomized, double-blind research with parallel arms. Research was carried out from 1st February 2023 to 30th May 2023 in the First Affiliated Hospital of Jinan University. Patients, with body mass index ranging from 30.6 to 51.2 kg/m^2^, scheduled for laparoscopic bariatric surgery, were randomly allocated to receive either propofol or ciprofol. For anesthesia induction and maintenance, participants in each group were administered propofol or ciprofol as the sedative agent. The mean cerebral blood flow velocities (CBFVm) were detected using transcranial doppler ultrasonography, and mean arterial pressure (MAP), heart rate and adverse events were recorded during surgery.

**Results:**

A total of 43 patients were ultimately included in this study (21 in the propofol group and 22 in the ciprofol group). Compared with baseline, patients receiving propofol group exhibited marked reductions in CBFVm at T_1_, T_2_, T_3_, T_4_, T_5_, T_7_, T_8_, T_9_ and MAP at T_1_, T_2_, T_3_, T_5_, T_8_ (*p* < 0.05), respectively. In contrast, ciprofol- administered participants demonstrated no significant change in CBFVm, and MAP only showed a significant decrease at T_1_ (*p* < 0.05). Although there was a certain difference in ΔCBFVm between the two groups, it was not statistically significant. Additionally, 13 patients (61.9%) in the propofol group experienced pain at the injection site, compared to only three patients (13.6%) in the ciprofol group (*p* = 0.001). Both groups experienced hypotension, respiratory depression, and bradycardia, but no significant differences were observed.

**Conclusion:**

This study found that, compared to propofol, ciprofol may be a better anesthetic choice for obese individuals receiving laparoscopic bariatric surgery, as it maintains CBFVm and hemodynamics more steadily, causes less injection discomfort, and demonstrates comparable safety and adverse event rates to those of propofol.

**Clinical Trial Registration:**

[https://www.chictr.org.cn/showproj.html?proj=187919], identifier [ChiCTR2300067801].

## Introduction

Since 1990, the prevalence of obesity among adults has more than doubled, while rates in adolescents have increased fourfold. By 2022, an estimated 2.5 billion people aged 18 years and older were classified as overweight, alongside over 390 million individuals aged 5–19 years. Within these groups, ~890 million adults and 160 million children or adolescents met the criteria for obesity (1, 2). Sleeve gastrectomy (SG) is a relatively fast and straightforward bariatric procedure that has demonstrated favorable outcomes in terms of weight reduction and improvement of obesity-related comorbidities. This surgery contributes to marked and long-term weight reduction, while also alleviating obesity-associated conditions, including metabolic syndrome, type 2 diabetes, and high blood pressure (3). Nevertheless, individuals with obesity continue to face a markedly greater likelihood of complications during the perioperative period compared with those of normal weight. The interaction between surgical positioning and pneumoperitoneum exerts remarkable impact on intracranial hemodynamics. The feet-down tilt position (re- Trendelenburg position) impedes cerebral venous return, while carbon dioxide pneumoperitoneum (intra-abdominal pressure: 12–15 mmHg) increases central venous pressure (CVP) by 8–12 mmHg through mechanical compression, thereby reducing cardiac output (CO) by 15%−25% (4, 5). Furthermore, preclinical animal studies have demonstrated that chronic inflammation and oxidative stress-mediated vascular endothelial damage impair cerebrovascular autoregulation (CA) function in obese mice (6, 7). These findings underscore the potential need to optimize perioperative cerebral hemodynamic management strategies for obese patients.

Propofol is widely administered for initiating and sustaining general anesthesia. In cases of severe obesity, the peripheral compartment volume is markedly enlarged (8). In addition, the dose-dependent vasodilatory effect of propofol can lower mean arterial pressure (MAP) up to 20%−30% of baseline (absolute values >25 mmHg) (9). In terms of cerebral circulation modulation, propofol achieved blood flow -metabolic coupling by decreasing the cerebral metabolic rate of oxygenation (CMRO_2_ decrease of 38%; IQR: 18%−46%) and regional cerebral blood flow (rCBF decrease of 28%; IQR: 10%−37%) (10). However, this mechanism may be weakened by the alternation of cerebrovascular pathology in obese patients.

Ciprofol (HSK3486) as a novel GABA-A receptor agonist with optimized pharmacokinetics by structural modification (2,6-diisopropylphenol cyclic phosphate prodrug). Ciprofol produces sedative efficacy at approximately 1/4 to 1/5 the dosage required for propofol, the median lethal dose (LD50) is one-fourth that of propofol (11), the incidence of injection pain is reduced from 28%−50% to 2.8%−5.3%, and the frequency of severe hypotension (MAP < 65 mmHg) during induction is reduced by approximately 20% compared with propofol (12–14). However, how ciprofol influences cerebrovascular auto-regulation capacity in individuals with obesity has not been clarified.

Existing studies of cerebral blood flow velocity analysis about propofol and ciprofol are mostly based on healthy volunteers, thus, the present research hypothesizes that ciprofol improves hemodynamic stability and reduces abnormal mean cerebral blood flow velocities (CBFVm) fluctuations in obese individuals receiving laparoscopic bariatric surgery. To provide evidence-based guidance and reference for the clinical application of ciprofol, this clinical trial was designed to validate its superior efficacy in preserving cerebral perfusion and maintaining hemodynamic stability.

## Methods

### Study design and ethics

This study was registered in the Chinese Clinical Trial Registry (ChiCTR2300067801) in 28th January 2023 (28/01/2023), and the ethics committee of the First Affiliated Hospital of Jinan University approved the study protocol (NO. KY-2022-129) in 22nd September 2022 (22/09/2022). This study was a parallel, double-blind, randomized controlled clinical trial. All the experiments were performed in accordance with relevant guidelines and regulations. All participants signed an informed consent.

### Inclusion and exclusion criteria

This research was carried out from 1st February 2023 to 30th May 2023 in the First Affiliated Hospital of Jinan University. Individuals with obesity receiving laparoscopic sleeve gastrectomy (LSG) or gastric bypass surgery qualified for enrollment in present research if they met the following criteria: (i) aged 18–65 years; (ii) had BMI > 28.5 kg/m^2^; (iii) American Society of Anesthesiologists (ASA) physical status classification I to III; (iv) comprehensive awareness of the study's objectives and implications; and (v) capacity to provide written informed consent.

The exclusion conditions were as follows: (i) inability to perform transcranial doppler (TCD) monitoring due to cerebral surgery; (ii) poorly controlled blood pressure fluctuations; (iii) history of cardiovascular and cerebrovascular diseases; (iv) degree III atrioventricular block; (v) hepatic or renal decompensation; (vi) requirement for dialysis therapy; (vii) presence of psychosocial disorders or cognitive impairment; and (viii) allergy to any drug in the medication regimen of this trial. (ix) involvement in other pharmaceutical clinical studies within 3 months; (x) additional scenarios where the investigator deemed the individual unsuitable for trial enrollment.

### Sample size

Based on preliminary results, the mean and standard deviation of the mean flow velocity [(group Propofol) 49.21 ± 13.20 vs. (group Ciprofol) 65.01 ± 15.25] were used as the main indicators, with a test power of 0.90 and a significance level of 0.05 specified in the analysis design. Accounting for a 15% attrition rate and maintaining equal group allocation, we determined that 22 participants per group were required, as calculated using PASS 15 software (NCSS, Kaysville, Utah).

### Randomization and blinding

Participant randomization was performed using the random number generator function in SPSS Statistics software (version 22.0). All assigned numbers were then sorted in ascending order, and patients were grouped based on this sorted sequence: those with odd numbers were allocated to the group Propofol, while those with even numbers were allocated to the group Ciprofol. The randomization personnel stored each patient's grouping information in a sealed envelope to maintain allocation concealment, (note: grouping was not determined by envelope drawing). Anesthesiologists were given syringes with solutions with codes according to the randomization order (to ensure the perioperative safety of patients, anesthesiologists who performed anesthesia were aware of the randomization). Personnel responsible for randomization and blinding processes were excluded from the follow-up assessments. Remaining study staff were masked to both group assignments and the identity of experimental medications. For allocation concealment, randomization outcomes were kept in sealed envelopes until study completion.

### Anesthesia procedures

Researchers conducted preoperative visits with the patients and assessed their basic information on the day before surgery. Eligible participants provided written informed consent and were asked to fast at least 6 h before surgery. Grouping was performed according to randomization. After verifying the patients' information on the day of surgery, a standard monitor was used to obtain the baseline values. A 20-gauge intravenous cannula was inserted into the upper dorsal vein to establish vascular access, then all participants administered 500 ml of sterile crystalloid solution before induction of anesthesia. All patients received intravenous dexamethasone (Runhong Pharmaceutical Group, China) 10 mg preoperatively, to prevent postoperative nausea and vomiting.

**Figure 1:**
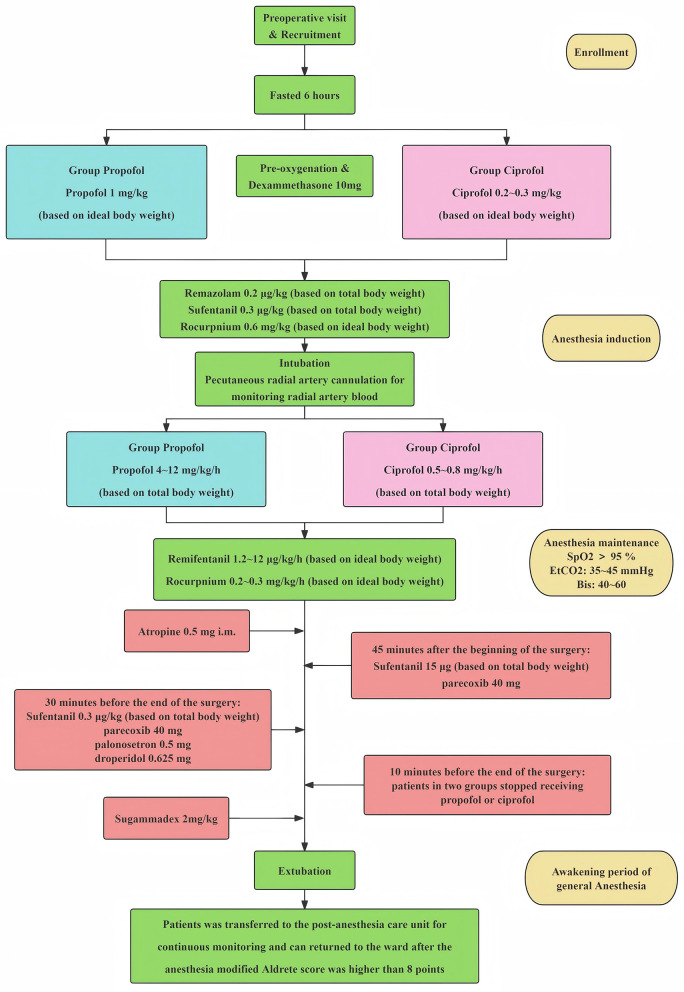
Anesthesia procedures.

The sedation procedure is illustrated in Figure 1. All patients underwent preoxygenation before anesthesia induction. For anesthesia induction, participants in group C administered 0.2–0.3 mg/kg (based on ideal body weight) of ciprofol (Haisco Pharmaceutical Group. China), and patients in group P received a propofol (Nhwa Pharmaceutical, China) dose of 1 mg/kg calculated using ideal body weight (BW) intravenously as sedative anesthetic. All patients received a dosage of 0.2 mg/kg determined by total BW of remazolam (Hengrui Pharmaceuticals, China), 0.3 μg/kg (using total BW for calculation) of sufentanil (Humanwell, China), and 0.6 mg/kg (using ideal BW for calculation) of rocuronium (Sigma-Aldrich, China) sequentially. All drugs were injected within 90 s. After 1 min of assisted mask ventilation, an endotracheal tube was placed, and atropine 0.5 mg was intramuscularly injected after intubation. During anesthesia maintenance, patients in group C received continued intravenous of ciprofol 0.5–0.8 mg/kg/h (based on total body weight) and patients in group P received continued intravenous of propofol 4–12 mg/kg/h (based on total body weight). All patients received intravenous of remifentanil (Humanwell, China) 1.2–12 μg/kg/h (according to ideal BW) and rocuronium 0.2–0.3 mg/kg/h (ideal BW as the reference). Patients in two groups received sufentanil 15 μg and parecoxib (Prizer, China) 40 mg intravenously 45 min after the beginning of the surgery and received sufentanil 0.3 μg/kg (based on ideal body weight), parecoxib 40 mg, palonosetron (China-gene, China) 0.5 mg and droperidol (Hualu Pharmaceutical Group. China) 0.625 mg 30 min before surgery conclusion. Oxygen saturation was kept above 95%, and end-tidal carbon dioxide was 35–45 mmHg throughout the surgery. The dose of the drugs was adjusted according to Blood Pressure (BP), heart rate (HR), and the patient's body movements, with the aim of maintaining a bispectral index (BIS) value between 40 and 60. Patients stopped receiving ciprofol (group C) or propofol (group P) 10 min before the end of surgery. Patients were intravenously injected with sugammadex sodium (Sigma-Aldrich, China) 2 mg/kg when spontaneous breathing was observed. After removal of the endotracheal tube, participants were sent to the post-anesthesia care unit (PACU) for continuous monitoring, and discharged to the ward after the modified Aldrete score exceeded nine points at PACU (15).

During surgery, phenylephrine (Harvest Pharmaceutical Group, China) 1 ml (0.1 mg/ml) was injected when invasive MAP lower than 65 mmHg and atropine (Xinghua Pharmaceutical Group, China) 0.5 mg was administered if HR fell below 50 beats per minute. CBFVm was measured using TCD, the hemodynamic index was recorded, and perioperative adverse events involving the respiratory or cardiovascular systems, which were similarly documented.

TCD standardized procedure: Upon the patient's entry into the operating room, they were instructed to rest quietly on the operating table for ~10 min to achieve hemodynamic stabilization. Subsequently, a fixed probe holder and TCD probe were secured, with the latter positioned at the temporal window to monitor the middle cerebral artery blood flow velocity. After adjusting the TCD probe to the angle yielding the optimal blood flow signal, the corresponding data were recorded as the T_0_ baseline values. Thereafter, TCD measurements were performed and recorded in accordance with the predefined time points. In cases where probe displacement occurred due to patient positional changes or other factors, resulting in attenuated TCD signals, the optimal signal angle was re-identified and the probe re-fixed prior to subsequent data collection.

### Outcome assessments and data collection

The primary outcomes of our research were the CBFVm monitored by TCD at different time points during surgery. Secondary outcome measures encompassed MAP, HR, and perioperative adverse events. Adverse events included cerebral ischemia (middle cerebral artery flow velocity < 20% of the basal value), injection pain, respiratory depression (SpO_2_ < 90% without oxygenation), hypotension (MAP < 65 mm Hg), bradycardia (HR: < 50 bpm), and postoperative delirium. A total of 11 time points were recorded throughout the perioperative period, including pre-anesthetic induction (T_0_), immediately following intubation (T_1_), 5 min after pneumoperitoneum (T_2_), 5 min after body position adjustment (rTP; T_3_), 15 min after body position adjustment (T_4_), 30 min after body position adjustment (T_5_), 45 min after body position adjustment (T_6_), 1 h after body position adjustment (T_7_), 5 min following pneumoperitoneum termination (T_8_), upon completion of surgery (T_9_), and 1 min after extubation (T_10_).

### Statistical analysis

Statistical analyses were conducted using GraphPad Prism software (version 8.3.0; San Diego, California). Continuous data were presented as mean ± SD, while categorical variables were summarized as counts and percentages [*n* (%)]. Normality of the dataset was assessed via the Kolmogorov–Smirnov test and histogram visualization. Normal distribution variables were compared using independent samples *t*-test, while not normal distribution variables were analyzed via the Mann-Whitney *U* test. Repeated measures data were evaluated with a repeated-measures analysis of variance (ANOVA). Categorical variables were examined using Pearson's chi-square test or Fisher's exact test, with statistical significance defined as a *p* value < 0.05.

## Results

### Participates inclusion and characteristics

**Figure 2:**
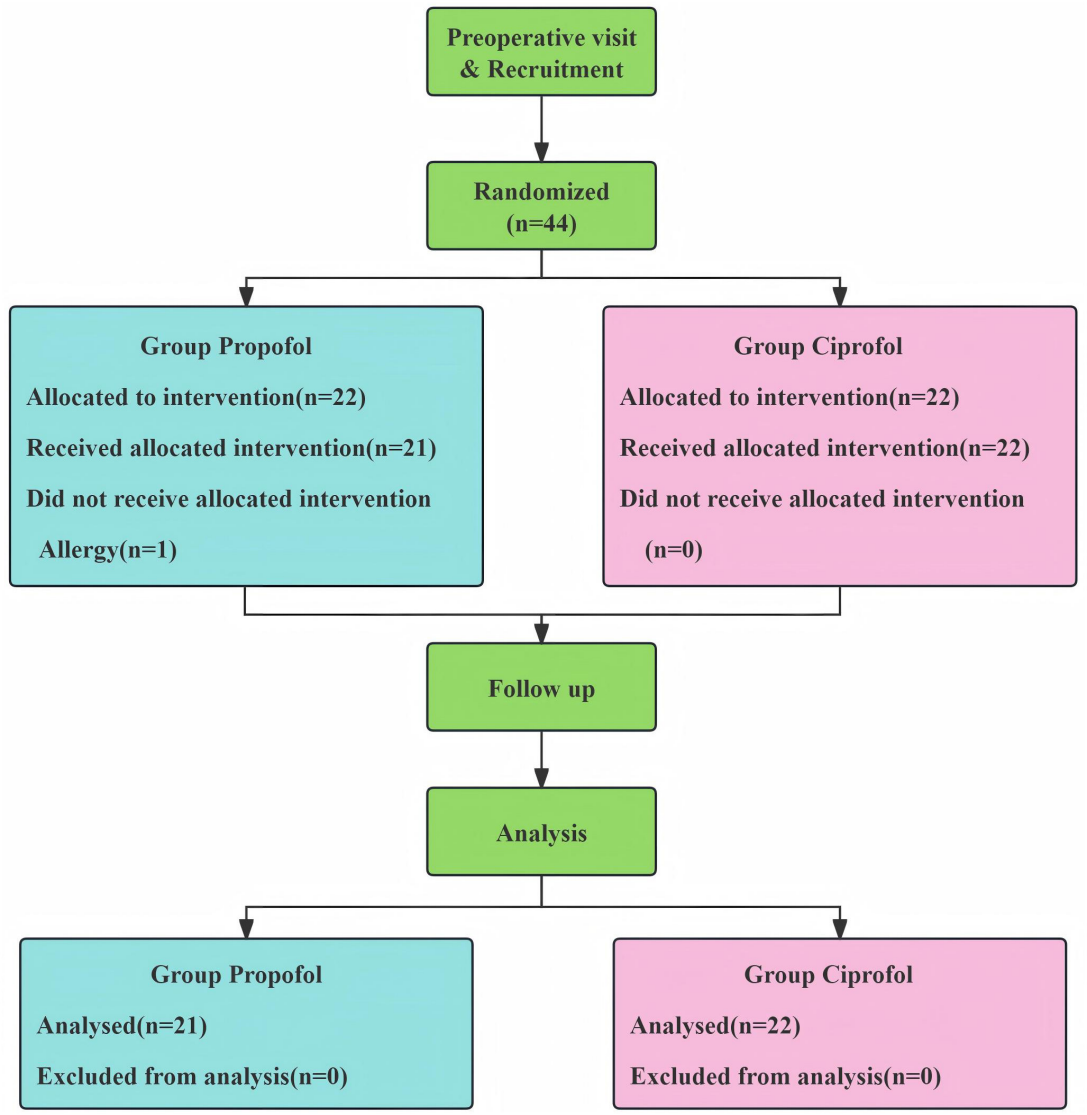
Patients enrollment diagram.

As shown in Figure 2, a total of 44 eligible patients were enrolled in this study and randomly assigned in equal numbers to the propofol group and the ciprofol group. One patient in the propofol group was withdrawn due to a severe allergic reaction to rocuronium bromide, resulting in 43 patients completing the study (21 in the propofol group and 22 in the ciprofol group). As shown in Table 1, there were no statistically significant differences between the two groups in terms of demographic characteristics, baseline CBFVm, MAP, and heart rate (*p* > 0.05), indicating well-balanced baseline characteristics and good intergroup comparability.

**Table 1:** Demographic and baseline characteristics of the participates.

**Age**	**All patients (*n* = 43)**	**Group propofol (*n* = 21)**	**Group ciprofol (*n* = 22)**	***p*-Value**
Age	32.63 ± 7.32	33.86 ± 7.65	31.45 ± 6.96	0.641
**Gender**				
Male	7 (16.3%)	4 (19.0%)	3 (13.6%)	0.631
Female	36 (83.7%)	17 (81.0%)	19 (86.4%)	
BMI (kg/m^2^)	39.16 ± 6.13	40.08 ± 6.34	38.28 ± 5.92	0.287
**ASA score**				
I	0	0	0	0.721
II	34 (79.1%)	16 (68.2%)	18 (81.8%)	
III	9 (20.9%)	5 (23.8%)	4 (18.2%)	
**Comorbidity**				
Hypertension	10 (23.3%)	5 (23.8%)	5 (22.7%)	0.572
Grade 1	9 (20.9%)	5 (23.8%)	4 (18.2%)	
Grade 2	1 (2.3%)	0	1 (4.5%)	
Grade 3	0	0	0	
Type 2 diabetes	5 (11.6%)	2 (9.5%)	3 (13.6%)	>0.999
OSA	7 (16.3%)	3 (14.3%)	4 (18.2%)	>0.999
**Baseline**				
CBFVm (cm/s)	64.86 ± 14.61	62.00 ± 10.20	67.59 ± 17.66	0.210
MAP (mmHg)	100.79 ± 11.41	101.57 ± 11.36	100.05 ± 11.67	0.253
Heart rate (bpm)	78.79 ± 13.73	79.10 ± 13.48	78.50 ± 14.28	0.889

Data are presented as mean ± SD or number (%). BMI, body mass index; ASA, American Society of Anesthesiologists; Hypertension grade (16), Grade 1 (140 mmHg ≦ Systolic blood pressure < 160 or 90 mmHg ≦ Diastolic blood pressure < 100 mmHg), Grade 2 (160 mmHg ≦ SBP < 180 or 100 mmHg ≦ DBP < 110 mmHg), Grade 3 (180 mmHg ≦ SBP or 110 mmHg ≦ DBP); OSA, obstructive sleep apnea; CBFVm, mean cerebral blood flow velocities; MAP, mean arterial pressure.

### Dynamic changes of CBFVm and MAP in the propofol and ciprofol groups

**Figure 3:**
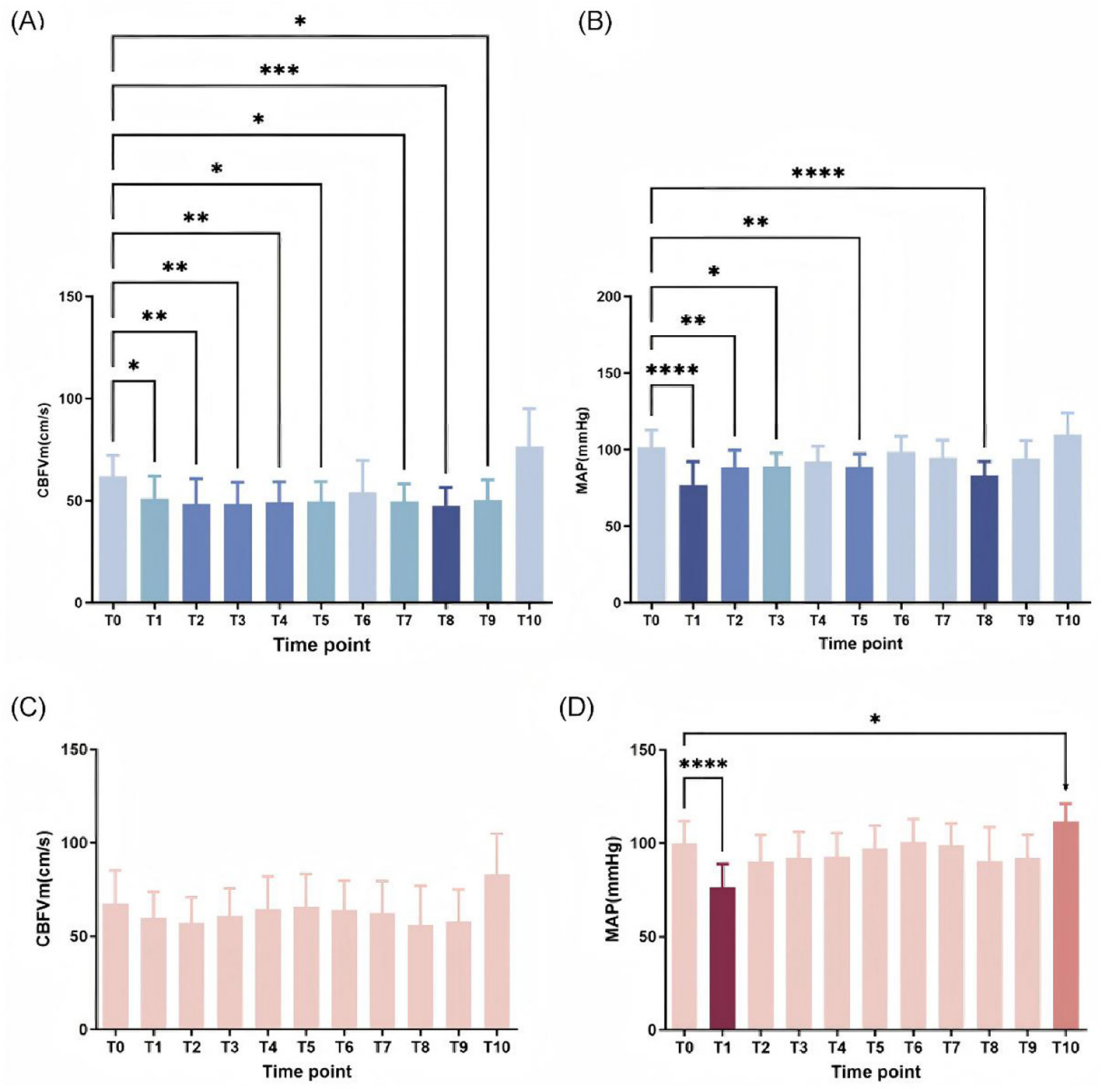
CBFVm and MAP of each time point was compared with baseline in Group P and Group C. **(A)** CBFVm of each time point was compared with T_0_ in Group P. **(B)** MAP of each time point was compared with T_0_ in Group P. **(C)** CBFVm of each time point was compared with T_0_ in Group C. **(D)** MAP of each time point was compared with T_0_ in Group C. CBFVm, mean cerebral blood flow velocities, MAP, mean arterial pressure. *p < 0.05 vs. T_0_, **p < 0.01 vs. T_0_, ***p < 0.001 vs. T_0_, ****p < 0.0001 vs. T_0_.

To study dynamic changes in cerebrovascular and systemic hemodynamics, we compared CBFVm and MAP at each time point relative to preoperative baseline (T_0_) between two groups. In the propofol cohort, both CBFVm and MAP exhibited significant decreases at most post-induction time points (*p* < 0.05), with maximal reductions observed at T_8_ (5 min after the end of pneumoperitoneum; Figures 3A,B). Notably, the temporal profiles of CBFVm and MAP declines were asynchronous: MAP reached its nadir immediately at T_1_ (post-intubation), whereas CBFVm remained suppressed and fluctuated at low levels before reaching its minimum at T_8_. This dissociation suggests that propofol may impair cerebrovascular autoregulatory function to a greater extent than its effect on systemic blood pressure. In contrast, the ciprofol group displayed transient MAP reduction exclusively at the first post-induction time point (T_1_, *p* < 0.05), with no significant changes in CBFVm throughout the observation period (Figures 3C,D), suggesting a mild effect of ciprofol on hemodynamic and cerebral hemodynamics.

### Comparison of CBFVm and MAP dynamics between two groups

**Figure 4:**
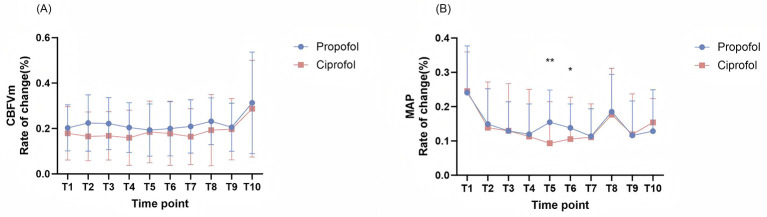
The rates of change of CBFVm and MAP between two groups. **(A)** The rates of change of CBFVm between two groups: There are no time points have significant differences between two groups, but show trend of difference in T2, T3, T4, T7, T8. **(B)** The rates of change of MAP between two groups: MAP has significant differences in T5, T6 between two groups. CBFVm, mean cerebral blood flow velocities; MAP, mean arterial pressure. *p < 0.05 vs. propofol group, **p < 0.01 vs. propofol group.

To systematically characterize the differential effects of ciprofol vs. propofol on cerebrovascular and systemic hemodynamics, we calculated the relative change indices (ΔCBFVm and ΔMAP) to quantify temporal variability compared to baseline measurements at each time point. For ΔMAP (Figure 4B), the ciprofol cohort exhibited notably less reductions than propofol cohort at T5 and T6 (*p* < 0.05), indicating attenuated systemic hemodynamic impact of ciprofol during these critical intervals. Regarding ΔCBFVm (Figure 4A), although no significant intergroup difference was observed in the reduction magnitude (*p* > 0.05), it is still apparent that the overall rate of change in CBFVm was lower among participants receiving ciprofol compared to the propofol-treated cohort.

### Incidence of adverse events

Safety profiles were evaluated throughout the study period, with adverse events (AEs) summarized in Table 2. In the group C, the most frequent AEs included hypotension (5/22, 22.7%), postoperative respiratory depression (5/22, 22.7%), and injection-site pain (3/22, 13.6%). In the group P, hypotension occurred in 10/21 patients (47.6%), postoperative respiratory depression in 3/21 (14.3%), injection-site pain in 13/21 (61.9%), and bradycardia in 1/21 (4.8%). Both groups of patients did not experience delirium. Statistically, no notable intergroup differences were observed in the occurrence of hypotension, respiratory depression, or bradycardia (*p* > 0.05). Notably, injection-site pain was 4.5-fold more common in the propofol group (61.9 vs. 13.6%, absolute risk difference: 48.3%), representing the most clinically relevant AE disparity between cohorts.

**Table 2:** Incidence of adverse events during induction.

**Perioperative complications**	**All patients (*n* = 43)**	**Group propofol (*n* = 21)**	**Group ciprofol (*n* = 22)**	***p*-Value**
Hypotension	15 (34.9%)	10 (47.6%)	5 (22.7%)	0.087
Respiratory depression	8 (18.6%)	3 (14.3%)	5 (22.7%)	0.477
Injection pain	22 (37.2%)	13 (61.9%)	3 (13.6%)	0.001^*^
Bradycardia	1 (2.3%)	1 (4.8%)	0	0.300

Data are presented as number (%). ^*^p < 0.05 vs. group propofol.

## Discussion

The global obesity pandemic has elevated bariatric surgery to a cornerstone of morbid obesity management, yet the perioperative period exposes obese patients to unique cerebrovascular risks, including baseline cerebral blood flow reduction and exacerbated hemodynamic lability (17). This vulnerability underscores the need for anesthetic agents that balance surgical efficacy with neurovascular stability. Ciprofol is a novel propofol analog, which has been found to be hemodynamically stable and can significantly reduce the incidence of adverse events in clinical practice (12–14, 18–22). Our study found that ciprofol maintained more stable middle cerebral blood flow velocities compared with propofol during laparoscopic bariatric surgery. Ciprofol was superior in terms of hemodynamic stability, and significantly reduced the incidence of injection pain.

Obesity increases patients' risk of developing cardiovascular and cerebrovascular diseases (2, 23). Intraoperative blood pressure fluctuations further elevate this risk in obese patients (24). Appropriate anesthetic selection during induction and maintenance, achieving optimal sedation depth and hemodynamic stability, can help reduce complications in this population (25). Previous studies have demonstrated that ciprofol offers rapid onset, quick recovery, and most importantly, superior hemodynamic stability (12–14, 18–22). Recent evidence confirms that ciprofol provides better sedation, fewer adverse events, and enhanced hemodynamic stability during general anesthesia induction in obese patients undergoing laparoscopic sleeve gastrectomy (26). Our findings align with these observations, showing that ciprofol maintained more stable blood pressure at various time points, with values closer to baseline levels compared to propofol. This advantage may stem from ciprofol's lower dosage requirement, thereby promoting better hemodynamic maintenance during surgery.

However, whether ciprofol provides superior cerebrovascular hemodynamic stability remains undetermined. TCD ultrasonography measures CBFVm, offering a noninvasive, simple, and rapid method to assess cerebral circulation (27–34). During surgery, real-time TCD monitoring enables anesthesiologists to adjust anesthetic dosages based on cerebral perfusion, thereby maintaining stable cerebral blood flow and potentially preventing cerebrovascular events. Notably, research has demonstrated an inverse correlation between BMI and cerebral blood flow in obese patients (17), resulting from complex interactions among metabolic dysregulation, dyslipidemia, and chronic inflammation. Furthermore, weight gain constitutes an independent risk factor for cognitive decline, which may be mediated by obesity-induced chronic cerebral hypoperfusion (35, 36).

Most intravenous anesthetics currently in use reduce cerebral blood flow (37). For obese patients undergoing laparoscopic bariatric surgery, selecting anesthetic agents with minimal cerebral hemodynamic impact is crucial. Our study revealed that ciprofol maintained relatively higher middle cerebral artery velocity compared to propofol in this population. Previous studies have found that pneumoperitoneum or the Trendelenburg position in laparoscopic surgery leads to further reduction in cerebral blood flow in patients (4, 5). Interestingly, our findings did not replicate this phenomenon. Conversely, ciprofol-sedated patients exhibited gradual recovery of cerebral blood flow velocity to baseline levels following pneumoperitoneum establishment and positional changes. The reduced propofol dosage required for adequate sedation with ciprofol administration contributed to more stable intraoperative blood pressure and cerebral perfusion. Since high-dose propofol may impair cerebral autoregulation (37), we propose that ciprofol better preserves cerebral arterial perfusion, thereby reducing anesthesia- and surgery-related cerebrovascular risks.

Beyond arterial blood flow velocity and hemodynamic parameters, we conducted a comprehensive analysis of adverse events to evaluate ciprofol's safety profile. While multiple studies have reported comparable incidences of injection pain, hypotension, respiratory depression, bradycardia, and postoperative delirium between propofol and its analogs, our findings revealed no significant intergroup differences in hypotension, respiratory depression, or bradycardia among obese patients undergoing laparoscopic bariatric surgery. Notably, neither group exhibited postoperative delirium, potentially attributable to the younger age profile (predominantly young-to-middle-aged adults) of our cohort, who generally demonstrate better tolerance to surgical and anesthetic stress. A striking difference emerged in injection pain incidence, with ciprofol demonstrating markedly lower rates (13.6%) compared to propofol (61.9%). Approximately three-fifths of patients experienced propofol-induced pain, with some recalling anesthesia induction as the most distressing perioperative experience. As a structural analog of propofol, ciprofol's incorporation of a cyclopropyl group and R-chiral center enhances its pharmacological and physicochemical properties, resulting in both greater potency and reduced injection pain relative to propofol. Collectively, the stable hemodynamic parameters, comparable adverse event profiles, and absence of postoperative delirium substantiate the safety of ciprofol for anesthesia management in obese patients undergoing laparoscopic bariatric procedures.

Our study had some limitations. First, CBFVm combined with cerebral oxygen saturation can better evaluate tissue metabolism in patients; however, this study did not monitor brain tissue oxygenation. Second, we observed the postoperative only in patients with postoperative cognitive changes. Long-term follow-up to assess neurological complications may better evaluate the effects of ciprofol on cognitive function. Finally, multicenter studies with larger sample sizes are required to confirm our findings. Future research should investigate the molecular mechanisms underlying ciprofol's ability to stabilize cerebral blood flow, which may provide a stronger theoretical basis for its clinical application.

## Conclusion

In this study, we demonstrate that ciprofol maintains more stable middle cerebral artery blood flow velocity and systemic hemodynamics compared to propofol during laparoscopic bariatric surgery in obese patients. Additionally, ciprofol exhibits a lower incidence of injection-site pain and favorable safety profile. These findings suggest ciprofol may serve as a superior anesthetic choice for laparoscopic bariatric surgery in obese patients, potentially enhancing perioperative patient comfort and safety.

## Data Availability

The original contributions presented in the study are included in the article/supplementary material, further inquiries can be directed to the corresponding author.

## References

[1] GBD 2021 Adult BMI Collaborators. Global, regional, and national prevalence of adult overweight and obesity, 1990-2021, with forecasts to 2050: a forecasting study for the global burden of disease study 2021. Lancet. (2025) 405:813–38. 40049186 10.1016/S0140-6736(25)00355-1 PMC11920007

[2] Okunogbe A Nugent R Spencer G Powis J Ralston J Wilding J. Economic impacts of overweight and obesity: current and future estimates for 161 countries. BMJ Glob Health. (2022) 7:e00973. doi: 10.1136/bmjgh-2022-009773 36130777 PMC9494015

[3] Gagner M Deitel M Erickson AL Crosby RD. Survey on laparoscopic sleeve gastrectomy (LSG) at the fourth international consensus summit on sleeve gastrectomy. Obes Surg. (2013) 23:2013–7. doi: 10.1007/s11695-013-1040-x 23912263

[4] Skytioti M Elstad M Sovik S. Internal carotid artery blood flow response to anesthesia, pneumoperitoneum, and head-up tilt during laparoscopic cholecystectomy. Anesthesiology. (2019) 131:512–20. doi: 10.1097/ALN.0000000000002838 31261258

[5] Yu J Park JY Hong JH Hwang JH Kim YK. Effect of pneumoperitoneum and Trendelenburg position on internal carotid artery blood flow measured by ultrasound during robotic prostatectomy. Clin Physiol Funct Imaging. (2022) 42:139–45. doi: 10.1111/cpf.12742 35018713

[6] Erdös B Snipes JA Miller AW Busija DW. Cerebrovascular dysfunction in Zucker obese rats is mediated by oxidative stress and protein kinase C. Diabetes. (2004) 53:1352–9. doi: 10.2337/diabetes.53.5.1352 15111506

[7] Phillips SA Sylvester FA Frisbee JC. Oxidant stress and constrictor reactivity impair cerebral artery dilation in obese Zucker rats. Am J Physiol Regul Integr Comp Physiol. (2005) 288:R522–30. doi: 10.1152/ajpregu.00655.2004 15514104

[8] Dong D Peng X Liu J Qian H Li J Wu B. Morbid obesity alters both pharmacokinetics and pharmacodynamics of propofol: dosing recommendation for anesthesia induction. Drug Metab Dispos. (2016) 44:1579–83. doi: 10.1124/dmd.116.071605 27481855

[9] Su H Eleveld DJ Struys MMRF Colin PJ. Mechanism-based pharmacodynamic model for propofol haemodynamic effects in healthy volunteers(?). Br J Anaesth. (2022) 128:806–16. doi: 10.1016/j.bja.2022.01.022 35249706

[10] Tagliabue S Lindner C da Prat IC Sanchez-Guerrero A Serra I Kacprzak M . Comparison of cerebral metabolic rate of oxygen, blood flow, and bispectral index under general anesthesia. Neurophotonics. (2023) 10:15006. doi: 10.1117/1.NPh.10.1.015006 36911206 PMC9993084

[11] Liao J Li M Huang C Yu Y Chen Y Gan J . Pharmacodynamics and pharmacokinetics of HSK3486, a novel 2,6-disubstituted phenol derivative as a general anesthetic. Front Pharmacol. (2022) 13:830791. doi: 10.3389/fphar.2022.830791 35185584 PMC8851058

[12] Liang Z Liu J Chen S Zhao X Chen G Xie Y . Postoperative quality of recovery comparison between ciprofol and propofol in total intravenous anesthesia for elderly patients undergoing laparoscopic major abdominal surgery: a randomized, controlled, double-blind, non-inferiority trial. J Clin Anesth. (2024) 99:111660. doi: 10.1016/j.jclinane.2024.111660 39426369

[13] Zou H Xi F Fu Y Xu J Zhang P Li D . Bispectral index-monitored anesthesia induction in older adults undergoing elective surgery: comparing ciprofol and propofol in a prospective, single-center, double-blind, randomized controlled study. Drug Des Devel Ther. (2024) 18:4993–5003. doi: 10.2147/DDDT.S484532 39525043 PMC11549918

[14] Liao M Wu XR Hu JN Lin XZ Zhao TY Sun H. Comparative effective dose of ciprofol and propofol in suppressing cardiovascular responses to tracheal intubation. Sci Rep. (2025) 15:1822. doi: 10.1038/s41598-025-85968-2 39805976 PMC11730606

[15] Chinese Society of Anesthesiology. Consensus of Chinese experts on post-anesthesia monitoring and therapy (2021 edition). J Clin Anesthesiol. (2021) 37:89–94.

[16] McEvoy JW McCarthy CP Bruno RM Brouwers S Canavan MD Ceconi C . 2024 ESC guidelines for the management of elevated blood pressure and hypertension. Eur Heart J. (2024) 45:3912–4018. 39210715 10.1093/eurheartj/ehae178

[17] Adepoju L Danos D Green C Cook MW Schauer PR Albaugh VL. Effect of high-risk factors on postoperative major adverse cardiovascular and cerebrovascular events trends following bariatric surgery in the United States from 2012 to 2019. Obes Relat Dis. (2023) 19:59–67. doi: 10.1016/j.soard.2022.08.014 36209030

[18] Li J Wang X Liu J Wang X Li X Wang Y . Comparison of ciprofol (HSK3486) versus propofol for the induction of deep sedation during gastroscopy and colonoscopy procedures: a multi-centre, non-inferiority, randomized, controlled phase 3 clinical trial. Basic Clin Pharmacol Toxicol. (2022) 131:138–48. doi: 10.1111/bcpt.13761 35653554 PMC9543620

[19] Teng Y Ou M Wang X Zhang W Liu X Liang Y . Efficacy and safety of ciprofol for the sedation/anesthesia in patients undergoing colonoscopy: phase IIa and IIb multi-center clinical trials. Eur J Pharm Sci. (2021) 164:105904. doi: 10.1016/j.ejps.2021.105904 34116176

[20] Luo Z Tu H Zhang X Wang X Ouyang W Wei X . Efficacy and safety of HSK3486 for anesthesia/sedation in patients undergoing fiberoptic bronchoscopy: a multicenter, double-blind, propofol-controlled, randomized, phase 3 study. CNS Drugs. (2022) 36:301–13. doi: 10.1007/s40263-021-00890-1 35157236 PMC8927014

[21] Chen X Guo P Yang L Liu Z Yu D. Comparison and clinical value of ciprofol and propofol in intraoperative adverse reactions, operation, resuscitation, and satisfaction of patients under painless gastroenteroscopy anesthesia. Contrast Media Mol Imaging. (2022) 2022:9541060. doi: 10.1155/2022/9541060 35935320 PMC9314164

[22] Zeng Y Wang DX Lin ZM Liu J Wei XC Deng J . Efficacy and safety of HSK3486 for the induction and maintenance of general anesthesia in elective surgical patients: a multicenter, randomized, open-label, propofol-controlled phase 2 clinical trial. Eur Rev Med Pharmacol Sci. (2022) 26:1114–24. 35253166 10.26355/eurrev_202202_28101

[23] Andolfi C Fisichella PM. Epidemiology of obesity and associated comorbidities. J Laparoendosc Adv Surg Tech A. (2018) 28:919–24. doi: 10.1089/lap.2018.0380 30010474

[24] De Jong A Verzilli D Chanques G Futier E Jaber S. Preoperative risk and perioperative management of obese patients. Rev Mal Respir. (2019) 36:985–1001. doi: 10.1016/j.rmr.2019.01.009 31521434

[25] Huschak G Busch T Kaisers UX. Obesity in anesthesia and intensive care. Best Pract Res Clin Endocrinol Metab. (2013) 27:247–60. doi: 10.1016/j.beem.2013.02.001 23731886

[26] Chi X Xu Y Li Q Xia K Fu Q. Efficacy and safety of ciprofol for the induction of general anesthesia in patients with obesity undergoing laparoscopic sleeve gastrectomy: a double-blind randomized, controlled study. PLoS ONE. (2025) 20:e0329005. doi: 10.1371/journal.pone.0329005 40705792 PMC12289008

[27] Aaslid R Markwalder TM Nornes H. Noninvasive transcranial Doppler ultrasound recording of flow velocity in basal cerebral arteries. J Neurosurg. (1982) 57:769–74. doi: 10.3171/jns.1982.57.6.0769 7143059

[28] Kofke WA Dong ML Bloom M Policare R Janosky J Sekhar L. Transcranial Doppler ultrasonography with induction of anesthesia for neurosurgery. J Neurosurg Anesthesiol. (1994) 6:89–97. doi: 10.1097/00008506-199404000-00004 7912125

[29] Fodale V Schifilliti D Conti A Lucanto T Pino G Santamaria LB. Transcranial Doppler and anesthetics. Acta Anaesthesiol Scand. (2007) 51:839–47. doi: 10.1111/j.1399-6576.2007.01355.x 17635391

[30] Bellapart J Fraser JF. Transcranial Doppler assessment of cerebral autoregulation. Ultrasound Med Biol. (2009) 35:883–93. doi: 10.1016/j.ultrasmedbio.2009.01.005 19329245

[31] Dagal A Lam AM. Cerebral autoregulation and anesthesia. Curr Opin Anaesthesiol. (2009) 22:547–52. doi: 10.1097/ACO.0b013e32833020be 19620861

[32] Vavilala MS Newell DW Junger E Douville CM Aaslid R Rivara FP . Dynamic cerebral autoregulation in healthy adolescents. Acta Anaesthesiol Scand. (2002) 46:393–7. doi: 10.1034/j.1399-6576.2002.460411.x 11952439

[33] Kincaid MS. Transcranial Doppler ultrasonography: a diagnostic tool of increasing utility. Curr Opin Anaesthesiol. (2008) 21:552–9. doi: 10.1097/ACO.0b013e32830edc0b 18784478

[34] Kincaid MS Souter MJ Treggiari MM Yanez ND Moore A Lam AM. Accuracy of transcranial Doppler ultrasonography and single-photon emission computed tomography in the diagnosis of angiographically demonstrated cerebral vasospasm. J Neurosurg. (2009) 110:67–72. doi: 10.3171/2008.4.17520 18821830

[35] Cournot M Marquié JC Ansiau D Martinaud C Fonds H Ferrières J . Relation between body mass index and cognitive function in healthy middle-aged men and women. Neurology. (2006) 67:1208–14. doi: 10.1212/01.wnl.0000238082.13860.50 17030754

[36] Toda N Ayajiki K Okamura T. Obesity-induced cerebral hypoperfusion derived from endothelial dysfunction: one of the risk factors for Alzheimer's disease. Curr Alzheimer Res. (2014) 11:733–44. doi: 10.2174/156720501108140910120456 25212912

[37] Slupe AM Kirsch JR. Effects of anesthesia on cerebral blood flow, metabolism, and neuroprotection. J Cereb Blood Flow Metab. (2018) 38:2192–208. doi: 10.1177/0271678X18789273 30009645 PMC6282215

